# Dual Biocide Behaviour of Quaternary Ammonium Functionalized Mesoporous Silica Nanoparticles Loaded with Thymus Essential Oil for Stone Conservation

**DOI:** 10.3390/nano15110866

**Published:** 2025-06-04

**Authors:** Federico Olivieri, Elena Orlo, Elodia Spinelli, Rachele Castaldo, Gennaro Gentile, Silvia Licoccia, Margherita Lavorgna, Marino Lavorgna

**Affiliations:** 1 Institute of Polymers Composites and Biomaterials, National Research Council of Italy, 80078 Pozzuoli, Italy; federico.olivieri@cnr.it (F.O.); rachele.castaldo@cnr.it (R.C.); licoccia@uniroma2.it (S.L.); 2 Department of Environmental, Biological and Pharmaceutical Sciences and Technologies, University of Campania “Luigi Vanvitelli”, 81100 Caserta, Italy; elena.orlo@unicampania.it (E.O.); margherita.lavorgna@unicampania.it (M.L.); 3 Department of Chemical Science and Technology, University of Rome Tor Vergata, 00133 Rome, Italy; elodia.spinelli@students.uniroma2.eu; 4 Institute of Polymers Composites and Biomaterials, National Research Council of Italy, 80055 Portici, Italy; marino.lavorgna@cnr.it; 5 Interdepartmental Centre for Nanosciences & Nanotechnologies & Instrumentation (NAST), University of Rome Tor Vergata, 00133 Rome, Italy

**Keywords:** mesoporous silica nanoparticles (MSNs), quaternary ammonium compounds, thymus essential oil, biocide properties, stone conservation

## Abstract

Mesoporous silica nanoparticles (MSNs) functionalized with silane quaternary ammonium compounds (SiQACs) were synthesized and utilized as carriers for thymus essential oil (TO), a green bio-antifouling agent. The synthesis of MSNs functionalized with SiQACs was carried out in a single step, with clear advantages in terms of simplicity of the process, high yield (94%) and saving of reagents and solvents for the MSN purification. After loading with TO, this innovative dual-action antifouling system was able to integrate the intrinsic biocidal properties of SiQACs with the release of TO from MSN pores, resulting in an engineered material with prolonged efficacy. The antifouling compounds incorporated into the nanoparticles accounted for 42% of the total weight. The biocidal performance was evaluated by monitoring the growth inhibition of *Chlorella sorokiniana*, a microalga commonly associated with stone biodeterioration. Additionally, these nanoparticles were embedded in a commercial silane-based protective coating and applied to tuff stone samples to assess their ability to mitigate biofilm formation over extended periods. Results demonstrated the system’s high potential for durable protection against microbial colonization and biofilm growth on stone surfaces.

## 1. Introduction

Outdoor stone-built cultural heritage is perpetually affected by biodeterioration, through the growth of biofilms, which aesthetically and structurally affect the monuments [1]. The factors promoting the damaging of cultural heritage monuments are extremely various, depending on external parameters such as temperature, light exposition, relative humidity, and intrinsic substrate characteristics, such as porosity, hydrophilicity, thermal conductivity and chemical composition [2]. Typically, monuments are affected by macroscopic organisms like plants, algae and cyanobacteria and these, releasing organic nutrients during their life cycle, induce the growth of infesting microorganisms, such as fungi, mosses and lichens [3]. Other issues correlated to the biofilm proliferation involve the penetration of filaments into the pores of the structure, which can induce mechanical fractures and the accumulation of atmospheric pollutants, due to the progressive modification of a stone’s characteristics, such as its local pH and water permeability.

Several commercially available antifouling products act against the infesting organisms by protecting the integrity of the substrate. More specifically, the provided protection can be intended as passive, when the system builds a local environment which repels the biofilm growth, or active, when the employed biocides kill the microflora. Passive protection is generally supplied by coatings with good affinity to the substrate and are able to improve the substrate hydrophobicity in order to retard the microorganisms’ growth [4]. In contrast, active protection is provided by biocides. Biocides often lack in long-lasting efficiency and/or are toxic and environmentally unsustainable [5,6,7]. Moreover, if a repetitive protective treatment on the stone is required, due to the short duration of the biocidal effect, the infesting organisms can develop a resistance to the employed products [8]. Additionally, some water-repellent coatings were found to interfere with the biocidal capability of the active agent, and the biocide application can interfere with the hydrophobicity of the substrate [9]. It is also to be said that water-repellent coatings show good effectiveness against algae and lichens, but not much against fungi [10], suggesting the fundamental role of the biocides for a comprehensive protection against all kinds of infesting organisms. Researchers are still working on new systems which offer a good compromise among all the required properties [11,12,13,14].

Among biocide compounds, quaternary ammonium compounds (QACs), which exchange ions between the positively charged groups of their structure and the cell membranes, are highly effective in inducing cell death [15]. In particular, long hydrocarbon chains bonded to nitrogen have exhibited very good biocidal behaviour [16,17,18,19]. This class of compounds has already been used for the protection of cultural heritage. For instance, QACs have been used for their biocidal effect on stones once loaded onto clay [20], and on wood [21]. However, the use of low molecular weight quaternary ammonium salts is limited by their toxicity [22,23], and thus their covalent grafting onto inorganic nanocarriers would represent a significant approach to mitigate their potential harmfulness.

Concerning natural compounds, the most commonly proposed alternatives to synthetic biocides are plant extracts and essential oils [24,25,26]. Their action depends on various factors, such the amount of active molecules and their chemical nature [27]. However, the widespread use of essential oils for biocidal applications is severely limited by their hydrophobic character and the presence of highly volatile compounds, particularly for outdoor applications, in which temperature and humidity play a key role in the optimization of a protective system. Nevertheless, a component of thymus essential oil, thymol, a monoterpenoid phenol, is highly water soluble and is one of the main active ingredients with biocidal effects of thymus essential oil [28,29]. Indeed, thymol interferes with the lipid bilayer of the cell membrane, changing its fluidity and permeability [30].

Starting from this background, our approach was aimed at designing a new highly effective and safe smart biocide system based on the dual effects of a silane quaternary ammonium compound grafted onto mesoporous silica nanoparticles (MSNs) and essential oils loaded into the pores of the functional MSNs. Indeed, MSNs are widely exploited as nanocarriers of active agents as they are very versatile, due to their high porosity and chemical stability, finding application in several fields such as drug delivery and cultural heritage [31]. Because of their high specific surface area, in stark contrast to non-porous nanoparticles, it is possible to load or graft high amounts of functional molecules/groups into their pores [32,33,34,35], increasing the durability of the active agents and preventing potential aggregation phenomena [32]. Moreover, they are efficiently dispersible in various polymer matrices, allowing the realization of functional nanocomposite coatings [31,32,33,34,36]. Following this approach, 3-trimethoxysilyl propyldimethyl octadecyl ammonium chloride (*TPOAC*) was covalently bonded into the structure of MSNs. A high yield-high throughput one-step synthesis of MSNs grafted with *TPOAC* (*MSN-SiQAC*) was optimized. In this way, the SiQAC moieties were grafted on large surface area nanoparticles able to exploit their function. The porosity of functionalized nanoparticles was further exploited to load thymus essential oil in order to maximize the biocide properties of the nanoparticles, and also to overcome the practical limitations of the poor dispersibility in water media of thymus essential oil. Given that algae of the genus *Chlorella* are among the most common pioneer microalgae that colonize stone monuments and contribute to their biodeterioration [37,38,39], the antifouling properties of the synthesized nanoparticles were further evaluated by exposing *Chlorella sorokiniana*, a unicellular green alga, to the effect of *MSN-SiQAC* loaded with TO. The growth of the algae was monitored over time to assess the effectiveness of the nanoparticles in inhibiting algal growth. Then, the nanoparticles were dispersed in a commercial protective silane/siloxane product for stone protection application. Then, the biofilm growth was monitored over 9 months and compared to an untreated stone.

## 2. Materials and Methods

### 2.1. Materials

Tetraethyl orthosilicate (*TEOS*), cetyltrimethylammonium bromide (CTAB), triethanolamine (TEAH3), 3-trimethoxysilyl propyldimethyl octadecyl ammonium chloride (*TPOAC*), chloroform, ethanol, hydrochloric acid (HCl, assay 36%) and thymus essential oil (TO) were purchased from Sigma–Aldrich (Milan, Italy).

*Chlorella sorokiniana* was provided by Algal Collection at University Federico II (ACUF—code strain 830).

The commercial product Achibuild CB ECO (Achibuild, dry content 60 wt%), a water-repellent organic-modified silane and siloxane-based water dispersion, was obtained from Achitex Minerva (Vaiano Cremasco, Italy).

Neapolitan tuff with density of 1.2 ± 0.3 cm^3^ and a porosity of 65.9 ± 1.7% was obtained from a local cave. The tuff specimens had dimensions of 5 × 5 × 2 cm^3^ and were washed with distilled water and dried until constant weight before the tests.

### 2.2. Synthesis of MSN-SiQAC

Modified mesoporous silica nanoparticles (*MSN-SiQAC*) were prepared through a modified high-yield procedure [40]. CTAB, TEAH_3_ and H_2_O were mixed in a 0.06:8:80 molar ratio for 1 h at 80 °C (pH ≈ 10). Then, *TEOS* and *TPOAC* in a weight ratio of 9:1 were added in the following sequence: *TEOS* was added first to induce its pre-hydrolysis, and *TPOAC* was added after 10 min. At this point, the synthesis was continued for 1.5 h. *MSN-SiQAC* was collected through filtration and washed with a 1.5 M ethanol/HCl (volume ratio 1:8) solution. Then, *MSN-SiQAC* was washed with bi-distilled water until neutrality and dried under vacuum overnight. For comparison, non-functionalized MSNs were synthesized with the same procedure, using 10 g of *TEOS* and without the addition of *TPOAC* [32,34].

### 2.3. Loading Thymus Essential Oil into MSN-SiQAC

First, 200 mg of *MSN-SiQAC* was kept under reduced pressure (50 mbar) overnight. TO (15 mg) was dissolved in chloroform (50 mg), and the solution was loaded into the nanoparticles in 5 equal additions. The TO quantity was selected on the basis of a quantification of the pore volume of *MSN-SiQAC* by nitrogen adsorption analysis, in order to fill completely the pores of the nanoparticles. The obtained nanoparticles were coded MSN-SiQAC-TO. The same procedure was followed with non-functionalized MSN, and these nanoparticles were coded MSN-TO.

### 2.4. Nanocomposite Coating and Film Preparation

MSN, *MSN-SiQAC* and MSN-SiQAC-TO were dispersed in the commercial Achibuild water dispersion. In detail, 0.5 wt% of each kind of nanoparticle was dispersed in the Achibuild dispersion through ultrasonication with a Sonics Vibracell ultrasonic processor (Newton, MA, USA) (500 W, 20 kHz), at 25% of amplitude for 10 min, with 15 s/15 s on/off cycles; the dispersion was kept in an ice-water bath during the treatment to avoid its over-heating. The percentage amount of nanoparticles was calculated on the dry content of Achibuild. The obtained dispersions were deposited in Petri dishes with 4.5 cm diameter to obtain 0.2 mm thick films by casting. These dispersions were also poured onto tuff stones. As a blank, a pristine Achibuild film and an Achibuild-treated stone were also produced.

### 2.5. Characterization

Morphological analysis of the synthetized nanoparticles was performed through Transmission Electron Microscopy (TEM) using a FEI Tecnai G12 Spirit Twin (LaB6 source) at 120 kV acceleration voltage (FEI, Eindhoven, The Netherlands). TEM images were collected on a FEI Eagle 4 k CCD camera. Before the analysis, nanoparticles were dispersed in ethanol through sonication using the previously cited Sonics Vibracell ultrasonicator at 25% of amplitude for 5 min and then collected on carbon-coated copper grids.

The textural properties of MSN and *MSN-SiQAC* nanoparticles were analyzed by N_2_ adsorption at 77 K using a Micromeritics 3Flex analyzer (Norcross, GA, USA). The specific surface area of the nanoparticles was determined from the linear part of the Brunauer–Emmett–Teller (BET) equation. Nonlocal density functional theory (NLDFT) was applied to the nitrogen adsorption isotherms to evaluate the pore size distribution of the nanoparticles. Prior to the analyses, all samples were degassed at 100 °C under vacuum (*P* < 10^−7^ mbar), and all the adsorption measurements were performed using high purity gases (>99.999%).

Fourier transform infrared (FTIR) spectroscopy in attenuated total reflectance (ATR) mode was performed by a PerkinElmer Spectrum One FTIR spectrometer (Waltham, MA, USA) using a resolution of 4 cm^−1^ and 16 scan collections. MSN, MSN-TO, *MSN-SiQAC*, MSN-SiQAC-TO and all the realized films (Achibuild, Achibuild-MSN, Achibuild-*MSN-SiQAC*, and Achibuild-MSN-SiQAC-TO) were tested.

Thermogravimetric analysis (TGA) was conducted on a Perkin Elmer Pyris Diamond TG/DTA (Waltham, MA, USA). TGA was performed on MSN, *MSN-SiQAC*, MSN-TO and MSN-SiQAC-TO by recording a 10 min isothermal scan at 100 °C and then heating up to 800 °C using a heating rate of 20 °C/min in an oxidative atmosphere.

The kinetics of TO release from the smart nanocarriers MSN-TO and MSN-SiQAC-TO were evaluated in water at 25 °C and at pH 7.0, with a nanoparticle concentration of 0.015 mg/mL. The release of TO was monitored by measuring at constant time intervals the TO concentration of the solution through UV–vis spectroscopy analysis using a Jasco V570 UV spectrophotometer (Jasco, Easton, MD, USA). TO calibration curves in water were previously collected. Morphological analysis of treated tuff stones after their exposition to antifouling tests was performed by Scanning Electron Microscopy (SEM) analysis, using a FEI Quanta 200 FEG SEM (FEI, Eindhoven, The Netherlands) in high-vacuum mode.

### 2.6. Antifouling Behaviour

MSN, MSN-TO, *MSN-SiQAC* and MSN-SiQAC-TO were exposed to the green microalga *Chlorella sorokiniana*. The alga was grown in sterile conditions using the Bold Basal Medium (BBM) [41], with constant agitation and illumination at 3000 lux. The algal cells were counted using a Bürker chamber. A total of 105 cells were then added to 1.5 mL of 1% low-melting agarose and plated onto pre-agarized Petri dishes (8.5 cm diameter) containing BBM with 2% agar. The plates were incubated at 3000 lux for 10 days to allow for algal growth. After 10 days, dispersions of MSN, MSN-TO, *MSN-SiQAC*, and MSN-SiQAC-TO in the algal medium (100 mg in 1.5 mL) were prepared and poured onto the surface of the Petri dishes. A negative control was also prepared by pouring only 1.5 mL of the BBM onto the surface of separate Petri dishes to observe the natural algal growth. The biocidal effect was monitored for 0, 3, 7 and 10 days by registering the optical image using a digital camera, 48 MP, ƒ/1.78 aperture. Treated tuff stones were completely buried in soil (humidity 100 RH%, temperature between 10 and 35 °C), leaving the upper surface exposed to air, in order to monitor microorganisms’ growth for 9 months.

## 3. Results and Discussion

MSNs functionalized with *TPOAC* were produced by a one-step high-yield/high-throughput synthetic protocol in the presence of the templating agent CTAB and the amine TEAH_3_. With this approach, *MSN-SiQAC* was obtained with a yield of about 94%. For yield calculation, taking into account the molar ratio between *TEOS* and *TPOAC* (9:1) and approximating the elemental composition of the final *MSN-SiQAC* to (9/10) SiO_2_ (1/10) (SiO_3/2_ (CH_2_)_3_N(CH_3_)_2_(CH_2_)_17_CH_3_Cl) (molecular weight 96.7932 g/mol, omitting in this way the contribution of terminal hydroxyl or alkoxy groups), the maximum theoretical product/reagent weight ratio that could be obtained by the reaction (*P*/*R_MAX_*) was found to be 0.4082, due to Equation (1):

(1) $${P/R}_{MAX}=\frac{{MW}_{MSN-SiQAC}}{0.9\;*\;{MW}_{TEOS}+0.1*\;{MW}_{TPOAC}}$$

Therefore, the yield *Y* of reaction was found by Equation (2):

(2) $$Y=\frac{\left(weight\;MSN-SiQAC\;/\;weight\;(TEOS+TPOAC)\;\right)}{{P/R\;}_{MAX}}$$

**Figure 1:**
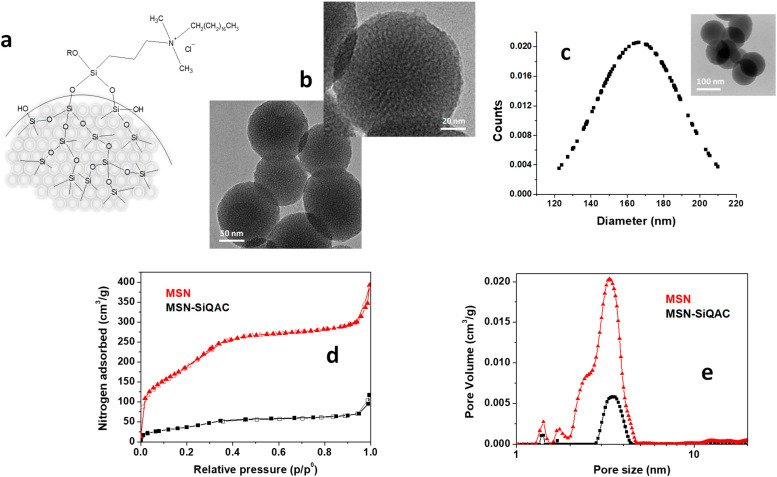
Scheme of *MSN-SiQAC* structure (**a**); TEM images of *MSN-SiQAC* (**b**); average diameter of *MSN-SiQAC* nanoparticles (**c**); nitrogen adsorption/desorption isotherms (**d**); and pore size distribution (**e**) of *MSN-SiQAC*.

The theoretical structure of *MSN-SiQAC*, according to reference [42], is reported in Figure 1a; OR may be either OH in the case of fully hydrolyzed silane molecules, or OCH3 in the case of partially hydrolyzed silane molecules. *MSN-SiQAC* particles have a homogeneous wormlike porosity (Figure 1b), and an average diameter, calculated by image analysis of several TEM images containing a total number of nanoparticles > 100, of 166 ± 39 nm, as shown in Figure 1c.

The nitrogen adsorption analyses of MSN and *MSN-SiQAC* nanoparticles are shown in Figure 1d,e. MSNs show a dual porosity distribution, with a major peak of porosity centered around 3.5 nm and a minor peak centered around 2.5 nm for MSNs, while the *MSN-SiQAC* nanoparticles exhibit only the porosity distribution peak centered around 3.5 nm. MSNs had a surface area of about 620 m^2^/g and total porosity of 0.57 cm^3^/g. The surface area and porosity of *MSN-SiQAC* decreased to 135 m^2^/g and 0.08 cm^3^/g, respectively. This considerable lowering is clearly related to the functionalization, which involved the external surface and the internal porosity of the nanoparticles.

However, the residual porosity of *MSN-SiQAC* was exploited to load TO. The surface area and porosity measurements were the basis for the determination of the TO amount loaded: since the TO density is 0.96 g/cm^3^, the maximum TO/*MSN-SiQAC* mass ratio was about 0.075. Then, for 200 mg of *MSN-SiQAC*, 15 mg of TO was dissolved in chloroform and loaded into *MSN-SiQAC*. Although the loading of TO onto the functionalized nanoparticles based on the dissolution of TO in chloroform is not an environmentally friendly process due to the use of the organic solvent, other impregnation methods based on supercritical fluids are potentially available [43,44,45]. Thus, although this is beyond the scope of the paper, the environmental sustainability of the proposed approach can be further improved.

**Figure 2:**
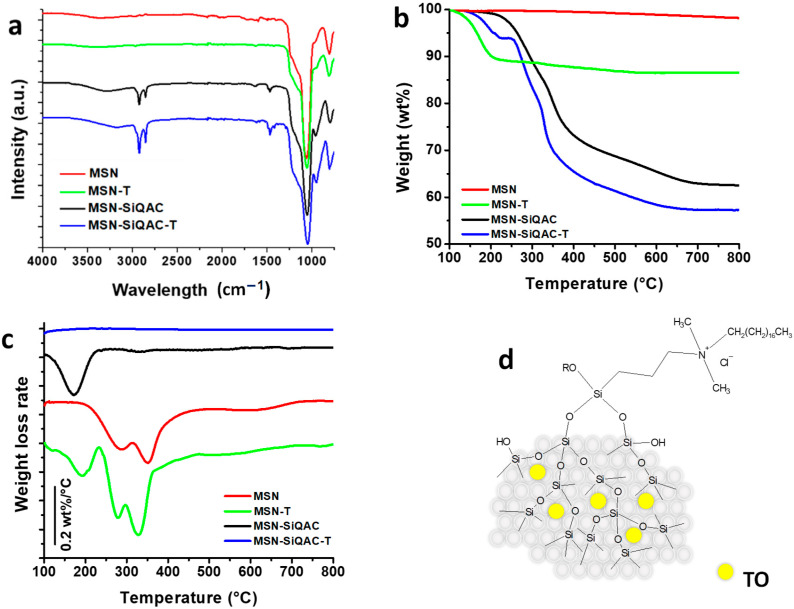
FTIR spectra (**a**), TGA traces (**b**), and DTG curves (**c**) of MSN, MSN-TO, *MSN-SiQAC* and MSN-SiQAC-TO; schematization of MSN-SiQAC-TO (**d**).

For comparison, the same amount of TO was loaded into non-functionalized MSNs. The efficiency of TO loading on functionalized and plain MSNs was then evaluated by FTIR and TGA analyses (Figure 2a,b). The FTIR analysis confirmed the efficacy in the MSN functionalization with *TPOAC*, with the presence of typical C-H stretching peaks between 2700 and 2950 cm^−1^, as well as a C-H bending peak at 1465 cm^−1^. On the other hand, the FTIR spectra did not show the presence of absorption bands clearly indicating the presence of TO, either in MSNs or *MSN-SiQAC*. However, the TO presence was confirmed by TGA analysis, which showed the presence of a weight loss peak associated with TO, which was evident in both MSN and *MSN-SiQAC* nanoparticles loaded with TO. This peak, well evidenced in the derivative weight loss curve (Figure 2c), is centered at about 175 °C for MSN-TO, and it shifted at a slightly higher temperature, about 195 °C, for MSN-SiQAC-TO.

TGA analysis allowed the quantification of the amount of grafted *TPOAC* and loaded TO (Figure 2b). The weight loss related to *TPOAC* functionalization is about 38%; TO loading in non-functionalized MSNs was about 13.5 wt%, and this value decreased to 5.3 wt% for *MSN-SiQAC*. The reduced amount of TO loaded into *MSN-SiQAC* with respect to MSNs was due to the considerable decrease in the inner porosity of *MSN-SiQAC* with respect to MSNs, as evidenced by gas adsorption analysis. However, the amount of TO loaded on functionalized *MSN-SiQAC* was very high because, comparing the pore volumes of MSNs and *MSN-SiQAC*, the amount of TO loaded in *MSN-SiQAC* should be lower than 25% of that loaded in pristine MSNs. Instead, the amount of TO loaded onto *MSN-SiQAC* was about 40% of the amount loaded onto plain MSN.

The biocidal activity of MSN-SiQAC-TO is due to the combined effect of *TPOAC* functionality, grafted on the nanoparticles surface, and TO, which can migrate from the mesopores to the external environment. The total amount of biocidal active molecules contained in these nanoparticles is about 42 wt%. In this way, the nanoparticles constitute a strong antifouling local spot when applied into a protective coating, and the capability of TO to diffuse from the pores of the nanoparticles expands the potential protected area around the nanoparticles.

**Figure 3:**
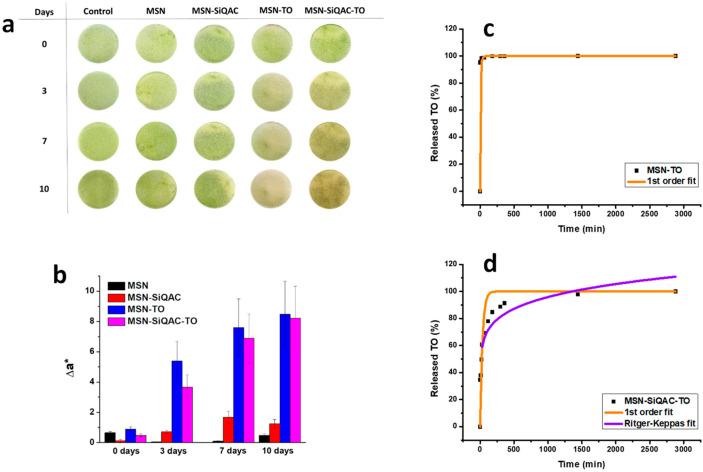
Evaluation of *Chlorella sorokiniana* growth in presence of MSN, MSN-TO, *MSN-SiQAC* and MSN-SiQAC-TO, at time 0, and after 3, 7 and 10 days (**a**). Δa* values calculated from the above-mentioned images (**b**). TO release kinetics and related fitting curves from MSN-TO (**c**) and MSN-SiQAC-TO (**d**).

To further evaluate the antifouling potential of the systems, nanoparticles were dispersed in the growth medium of *Chlorella sorokiniana* and applied to a solid medium where the algal cells had been cultured for 10 days. Figure 3a presents optical images of *Chlorella sorokiniana* treated with MSN, MSN-TO, *MSN-SiQAC*, and MSN-SiQAC-TO at time 0, and after 3, 7, and 10 days. The algal growth was inhibited after 3 days of exposure to MSN-TO and MSN-SiQAC-TO, with the biocidal effect becoming more pronounced after 7 days of exposure to MSN-TO and 10 days of exposure to MSN-SiQAC-TO. In contrast, MSN and *MSN-SiQAC* showed no significant changes during the observation period, with no noticeable differences compared to the control. Colorimetric analysis has been used as a method to quantitatively evaluate the growth of algae [46,47,48]. In this work, with the aim of performing a qualitative evaluation of the *Chlorella sorokiniana* growth, we performed a colorimetric analysis on the optical images using the CIELAB color system, which represents the quantitative relationship of colors on three axes. The L* value indicates lightness and a* and b* are chromaticity coordinates. In particular, the a* value indicates red–green component of a color, where +a* (positive) and −a* (negative) indicate red and green values, respectively. Therefore, the changes in the growth of green algae, as shown in Figure 3a, can be evaluated by comparing the Δa* values related to the single images of the growth media. The calculation of Δa* was performed by considering an average on three measures carried out on circular areas of 4 cm diameter in the Petri dishes, with the control used as a blank. A more pronounced increase in the a* value corresponds to a more pronounced disappearance of the green component, which is an indication of the amount of grown algae. The Δa* values shown in Figure 3b confirm that MSN-TO exhibited the best-performing antifouling capability, also at shorter test times, with respect to the other nanoparticles. Nevertheless, MSN-SiQAC-TO reached comparable effect to MSN-TO after 10 days of observation time.

Hence, mitigation of algae growth was significantly promoted by TO, and this effect was more pronounced when TO was loaded onto functionalized *MSN-SiQAC*, possibly due to a slower TO release kinetics from MSN-SiQAC-TO nanoparticles. These slower release kinetics of TO from MSN-SiQAC-TO in comparison to MSN-TO were confirmed by release tests in water, whose results are shown in Figure 3c and 3d, respectively, where the released amount of TO was normalized to the different amounts of TO loaded onto plain MSN and *MSN-SiQAC*. As shown, the TO release rate from MSN-SiQAC-TO is considerably slower than release rate from MSN-TO. In detail, the TO release from MSN-TO reached 95% after 10 min, while the same amount of TO was released from MSN-SiQAC-TO after about 24 h.

The release mechanisms of TO from MSN-SiQAC-TO and from MSN-TO were analyzed using typical drug release kinetic models [49]. In detail, experimental TO release curves were analyzed using several model-dependent approaches, including zero-order, first-order, Higuchi and Ritger–Peppas or Korsmeyer–Peppas regression models. Best results were obtained with the first order and the Ritger–Peppas regression models.

In particular, the TO release from MSN-TO was well fitted only with the first-order regression model, shown in Figure 3c. The R^2^ value for this fitting procedure was 0.997, and the k_1_ coefficient was 1.55 × 10^−3^ min^−1^. The first-order mechanism well describes the release of water-soluble active agents, such as TO, from porous matrices [50]. Indeed, other models, including the Ritger–Peppas regression model, gave a R^2^ value (<0.6), which is significantly lower than the R^2^ value associated with the first-order regression.

The release mechanism of TO was significantly modified when TO was loaded onto *MSN-SiQAC* nanoparticles. Indeed, as shown in Figure 3d, consistent with the already evidenced decrease in the TO release rate, the TO release mechanism was not in good agreement with the first-order regression mechanism (R^2^ = 0.808; k_1_ = 1.14 × 10^−2^ min^−1^). A better R^2^ value (R^2^ = 0.870) was obtained with the Ritger–Peppas regression model. With this regression model, the exponent factor *n* was found to be 0.138, indicating a Fickian diffusion release mechanism, already evidenced for the release of other active agents from engineered MSNs [51].

**Figure 4:**
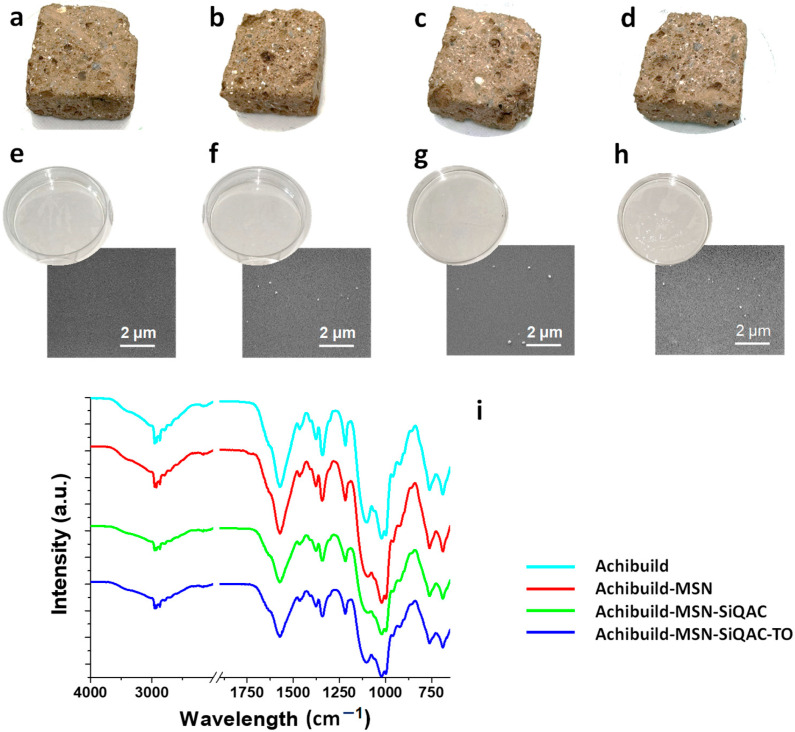
Optical images of tuff stone samples treated with Achibuild (**a**), Achibuild-MSN (**b**), Achibuild-MSN-TO (**c**), and Achibuild-MSN-SiQAC-TO (**d**) dispersions; films and corresponding SEM images of Achibuild (**e**), Achibuild-MSN (**f**), Achibuild-MSN-TO (**g**), and Achibuild-MSN-SiQAC-TO (**h**). FTIR spectra of the films (**i**).

To further confirm the antifouling behaviour of the combined SiQAC functionalization and TO loading, tuff stones were impregnated with the commercial protective product Achibuild, loaded with MSN, *MSN-SiQAC* and MSN-SiQAC-TO. It is worth noting that the limitations in loading free amounts of oil in the water-based Achibuild dispersion was overcome by embedding the TO in MSN-SiQAC-TO; the nanoparticles have, therefore, a key role in the embedding of essential oil into the coatings. For comparison, a tuff sample was also impregnated with pristine Achibuild. Images of tuff stone samples treated with the different dispersions are shown in Figure 4a–d. Films made by the same dispersions were also realized. In Figure 4e–h, the realized film and the corresponding SEM images of the film surfaces are shown. As shown by SEM analysis, MSNs have high chemical affinity to the siloxane groups of Achibuild, allowing an excellent dispersion of the nanoparticles in the films. The FTIR analysis (Figure 4i) did not show substantial differences among the films; also in this case, due to the chemical similarity between the loaded MSNs and the siloxane matrix, there were no substantial modifications of the FTIR signals.

**Figure 5:**
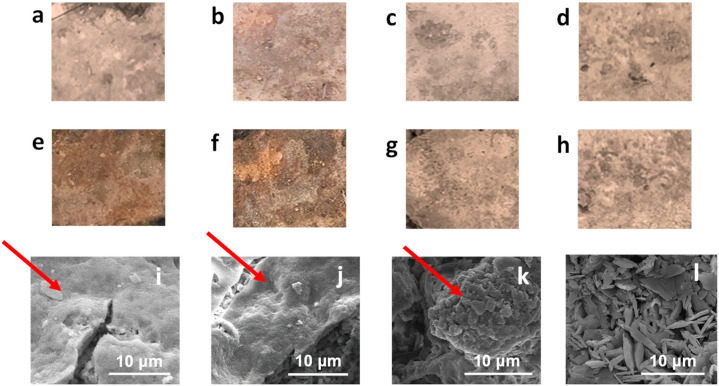
Surfaces of treated tuff stone samples before and after 9 months of biofilm growth by soil burial leaving the upper surface exposed to air. Optical images of unaged stones treated with Achibuild (**a**), Achibuild-MSN (**b**), Achibuild-MSN-TO (**c**), and Achibuild-MSN-SiQAC-TO (**d**) dispersions; optical images of stones treated with Achibuild (**e**), Achibuild-MSN (**f**), Achibuild-MSN-TO (**g**), and Achibuild-MSN-SiQAC-TO (**h**) dispersions after 9 months of soil burial; SEM images of Achibuild (**i**), Achibuild-MSN (**j**), Achibuild-MSN-TO (**k**), and Achibuild-MSN-SiQAC-TO (**l**) dispersions after 9 months of soil burial.

Tuff stones were buried in soil for 9 months leaving the upper surface exposed to air, and optical images of the sample surfaces were collected before and after the test (Figure 5a–h). The optical images showed a darkening of the substrates. This variation appeared more evident in the Achibuild and Achibuild-MSN samples; therefore, the addition of biocidal substances seems to improve the resistance of the stones to aggressive species. After 9 months, the samples were removed from the soil and their surface morphologies were evaluated by SEM analysis (Figure 5i–l). The collected micrographs clearly evidenced the presence of biofilms, marked with red arrows in Figure 5i–k, on samples treated with Achibuild (Figure 5i) and Achibuild-MSN (Figure 5j), as well as, partially, on the sample treated with Achibuild-MSN-TO (Figure 5k). The best results were obtained for Achibuild-MSN-SiQAC-TO (Figure 5l), whose surface was found to be very clean, with only some small spots of biofilm growth.

Therefore, all the results demonstrated the efficacy of the approach proposed here, which is based on the exploitation of mesoporous nanocarriers functionalized with a quaternary ammonium salt and loaded with thymus essential oil, resulting in a dual biocide capability. The embedding of TO overcame the limitations in using a hydrophobic substance for outdoor applications, and the quaternary ammonium salt grafted onto the nanoparticles decelerated the TO release from the pores, bypassing the issues related to its high volatility. Moreover, the long alkyl chain of SiQAC attached to the nanoparticles exhibited its own biocidal behaviour, leading to the realization of a nanomaterial with long-lasting effectiveness that is based on a dual antifouling system.

## 4. Conclusions

A new nanocarrier for antifouling applications was realized by functionalizing mesoporous silica nanoparticles with a quaternary ammonium long alkyl chain compound and loading them with thymus essential oil. The synthesis of silica nanoparticles functionalized with the selected quaternary ammonium long alkyl chain compound was carried out in a single step, with clear advantages in terms of the simplicity of the process, its high yield (94%) and savings in reagents and solvents used for the MSN purification. The protective action of the as-realized nanoparticles was based on a double effect: a direct contact protection, supplied by the quaternary ammonium salt; and a secondary effect ascribed to the release of thymus essential oil. The effectiveness of the synthesis of the modified mesoporous silica nanoparticles, namely *MSN-SiQAC*, was proved by FTIR, TGA, TEM, and SEM, as well as surface area and porosity analyses. The results showed the high efficiency of the optimized one-step synthesis in terms of yield and dimensional homogeneity. FTIR and TGA were also exploited to evaluate the effectiveness of the TO loading, which was not dramatically affected by the significant reduction in surface area of *MSN-SiQAC* with respect to non-functionalized MSN. In particular, the total biocidal amount loaded and grafted to *MSN-SiQAC* reached 42 wt%. Finally, the realized nanoparticles were tested as an antifouling agent by evaluating the growth of the green alga *Chlorella sorokiniana* on a solid medium in the presence of the nanoparticles. Furthermore, the nanoparticles were embedded in a commercial product that utilizes antifouling nanocomposites and is used to impregnate tuff stones and prevent the growth of films. The growth of the alga was monitored by optical images, and the TO release kinetics were also evaluated by UV–vis spectroscopy. The tuff samples were characterized by FTIR and SEM analysis, while the stones were exposed to the external environment to monitor the bio-organisms’ growth in the presence of the biocidal loaded nanoparticles. The results show the effective TO release from the nanoparticles and increased protection of the substrates with the use of the functionalized nanoparticles. These results reveal the high potential of these dual-action antifouling nanoparticles for different applications and substrates.

## Data Availability

The raw data supporting the conclusions of this article will be made available by the authors on request.
